# Acknowledgment to Reviewers of *International Journal of Neonatal Screening* in 2021

**DOI:** 10.3390/ijns8010010

**Published:** 2022-01-26

**Authors:** International Journal of Neonatal Screening Editorial Office

**Affiliations:** MDPI AG, St. Alban-Anlage 66, 4052 Basel, Switzerland

Rigorous peer-reviews are the basis of high-quality academic publishing. Thanks to the great efforts of our reviewers, *International Journal of Neonatal Screening* was able to maintain its standards for the high quality of its published papers. Thanks to the contribution of our reviewers, in 2021, the median time to first decision was 18 days and the median time to publication was 44 days. The editors would like to extend their gratitude and recognition to the following reviewers for their precious time and dedication, regardless of whether the papers they reviewed were finally published:
Aaron GoldenbergLisa M. GehtlandAlessandra EvaLuca BrunelliAlex R. KemperM. Elske Van Den Akker-van MarleAlexander AsamoahMaartje BlomAllan Meldgaard LundMalcolm DonaldsonAmy CunninghamMarelle J. BouvaAmy E. Wiberley-BradfordMaria ContaldoAmy GaviglioMaria Francisca CoutinhoAna Argudo RamírezMariaAnna MessinaAngela ScheuerleMarie AudrainAnita BoelenMark R. De HoraAnna SedivaMarleen JansenAntonella OlivieriMatthew HendersonAntonia RibesMay Ee PngArindam BhattacharjeeMei BakerBaba InusaMichele CagganaBeth A. TariniMiguel Angel Alcántara-OrtigozaBradford TherrellMimi S. KimCan FiciciogluMirjam D. Van Der BurgCarla CuthbertNatalya KashirskayaCarmencita PadillaNatasha HeatherCarol JohnsonNenad BlauCatharina SchuetzNikolas BoyChloe Miu MakNobuyuki IshigeColin KennedyNorma P. TavakoliCynthia F. HintonPamela A. Harris-HamanDavid CheillanPaolo CavorettoDavid WengerPatrice K. HeldDawn A LaneyPatrick DucoroyDeborah MarsdenPatrick JayDebra FreedenbergPaul Van TrotsenburgDenise Margaret HarrisonPaula J. WatersDietrich MaternPeter ClementsEduardo TizzanoPéter MonostoriEnzo RanieriPeter SchielenErnest M. PostPhilip J. YoungFabian HauckPim W. J. VergeerFlorian Sebald EichlerPin-Wen ChenFrançois BoemerR. Rodney HowellFrancois EyskensRajendra SinghFrancyne KubaskiRalph FingerhutFrömmel ClaudiaReinson KaritGeorge E. HogansonRichard B. ParadGeorge TavartkiladzeRobert CurrierGo TajimaRoberto GiuglianiGustavo J. C. BorrajoRolf ZetterströmGuy Van VlietRose E. MaaseHanneke A. HaijesRui VitorinoHarvey LevySamantha A. VerganoHisahide NishioScott D. GrosseIsabelle Durand-ZaleskiScott ShoneJ. Gerard LoeberSerena GasperiniJames B. GibsonSerwet DemirdasJames PittShozo TomonagaJennifer TaylorShunji TomatsuJim BonhamStephan KempJoe OrsiniStuart MoatJosé A. CochoStuart Paul AdamsJovanka KingSvetozár DluholuckýKanshi MinamitaniTudor Lucian PopKarin EngströmUlrika Von DöbelnKarl R. WhiteVěra FrankováKatarina Trebušak-PodkrajšekVeronica WileyKate E. ArmstrongVeroniqa LundbäckKaterina S. KuceraVijaya KnightKatja EggermannVineet DhimanKazumichi FujiokaVito TerlizziKendra JonesWuh-Liang HwuKenji YamadaYvonne A. DanielKonstantinos PetritisYvonne Kellar-GuentherLeah HechtZenon PogorelićLennart HammarströmZheng Feei Ma


